# Patient and pathological predictors of management strategy for malignant polyps following polypectomy: a systematic review and meta-analysis

**DOI:** 10.1007/s00384-022-04142-6

**Published:** 2022-04-08

**Authors:** Andrew P. Zammit, Nicholas J. Lyons, Mark D. Chatfield, John D. Hooper, Ian Brown, David A. Clark, Andrew D. Riddell

**Affiliations:** 1 Faculty of Medicine, University of Queensland, Brisbane, QLD Australia; 2 Mater Research Institute, The University of Queensland, Brisbane, QLD Australia; 3 Envoi Specialist Pathologists, Brisbane, QLD Australia; 4 Royal Brisbane and Women’s Hospital, Brisbane, QLD Australia; 5 Faculty of Medicine and Health, University of Sydney and Surgical Outcomes Research Centre (SOuRCe), Sydney, NSW Australia; 6 St Vincent’s Private Hospital Northside, Brisbane, QLD Australia; 7 Redcliffe Hospital, Redcliffe, QLD Australia

**Keywords:** Malignant polyp, Colorectal cancer, Cancer in adenoma, Polypectomy, Surgical resection

## Abstract

**Purpose:**

Malignant polyps present a treatment dilemma for clinicians and patients. This meta-analysis sought to identify the factors that predicted the management strategy for patients diagnosed with a malignant polyp.

**Methods:**

A literature search was performed following the Preferred Reporting Items for Systematic Reviews and Meta-Analyses (PRISMA) and the Cochrane Collaboration prognostic studies guidelines. Reports from 1985 onwards were included, data on patient and pathological factors were extracted and random effects meta-analysis models were used.

**Results:**

Fifteen studies were included. Seven studies evaluated lymphovascular invasion (LVI). The odds of surgery were significantly higher in malignant polyps with LVI (OR 2.20, 95% CI 1.36–3.55). Ten studies revealed the odds of surgery were significantly higher with positive polypectomy margins (OR 8.09, 95% CI 4.88–13.40). Tumour differentiation was compared in eight studies. There were significantly lower odds of surgery in malignant polyps with well/moderate differentiation compared with poor differentiation (OR 0.31, 95% CI 0.21–0.46). There were non-significant trends favouring surgical resection in younger patients, males and Haggitt 4/Kikuchi Sm3 lesions. There was considerable heterogeneity in the meta-analyses for the variables age, gender, polyp morphology and Haggitt/Kikuchi level (*I*^2^ > 75%).

**Conclusion:**

This meta-analysis has demonstrated that LVI, positive polypectomy resection margins, and poor tumour differentiation significantly predict malignant polypectomy patients who underwent subsequent surgery. Age and gender were important factors predicting management, but not consistently across studies, whilst polyp morphology and Haggitt/Kikuchi levels did not significantly predict the management strategy. Further research may assist in understanding the management preferences.

## Introduction

### Background

Colorectal adenocarcinoma is one of the most commonly diagnosed cancers within Australia [1] and worldwide [2]. The adenoma-carcinoma sequence is described as a stepwise progression from normal mucosa, to adenoma, to invasive carcinoma [3]. Early in the process of carcinogenesis, malignancy may be restricted to the polyp, known as a malignant colorectal polyp, or malignant polyp. Malignant polyps are defined as any macroscopically complete endoluminal resection of an adenoma that contains a focus of adenocarcinoma, invading through the muscularis mucosa into the submucosa [4, 5]. Malignant cells must be seen to be invading into the submucosa (thus excluding intramucosal carcinoma). Cancers invading beyond the submucosa (i.e. T2 or higher) are no longer considered malignant polyps. Overall, the literature reports that between 0.75% and 5.6% of all colorectal adenomas contain submucosally invasive adenocarcinoma [6]. Furthermore, an increased incidence of malignant polyps has been noted with the commencement of bowel cancer screening programmes [7].

**Fig. 1:**
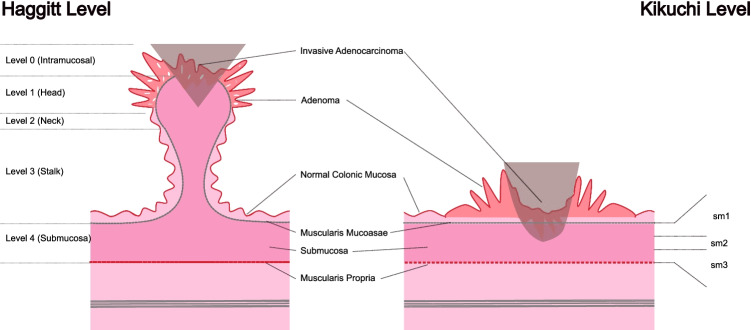
Haggitt and Kikuchi description of level of invasion. Level of invasion as described by Haggitt for a pedunculated polyp (left) and by Kikuchi for a sessile polyp (right). Submucosa (sm) 1 carcinomas invade through the muscularis mucosae to a depth of 200 to 300 μm, Sm2 lies between Sm1 and Sm3, and Sm3 approaches the muscularis propria [4, 7]

The difficulty in determining the optimal management strategy for malignant polyps lies in the assessment of risk of residual disease in the bowel wall or metastatic lymphatic spread. One of the earliest described factors predicting risk of lymphatic spread was depth of invasion, sub-staged into levels by Haggitt concerning pedunculated polyps [5] and Kikuchi for sessile polyps [8]. Haggitt and Kikuchi levels are shown in Fig. 1. Haggitt reported a significant difference in the rate of adverse events—those being adenocarcinoma spread to draining lymph nodes or mortality due to colorectal cancer—for Haggitt level 4 polyps [5]. Kikuchi reported increased risk of lymph node involvement or an involved resection margin with submucosa (sm) 2 or sm3 level malignant polyps compared with sm1 level malignant polyps [8].

In addition to Haggitt and Kikuchi levels, other pathological factors have been identified resulting in higher rates of residual disease or spread to adjacent lymphatics following polypectomy of a malignant polyp. These include poor tumour differentiation, tumour budding, presence of lymphovascular invasion (LVI) and close or involved polypectomy margins and are reflected in guidelines developed by societies of colorectal surgeons, gastroenterologists and pathologists [6, 9, 10]. The Association of Coloproctology of Great Britain and Ireland (ACPGBI), American Gastroenterological Association (AGA) and Japanese Society for Cancer of the Colon and Rectum (JSCCR) recommendations are summarised in Table 1 [6, 9, 11].

**Table 1:** Summary of guidelines for consideration for resection

	ACPGBI [6]	AGA [9]		JSCCR [11]
		Pedunculated	Sessile	
Margins	< 1 mm (*High risk)* 1–2 mm (*Intermediate risk)*	< 1 mm	Involved cautery margin	Resection not required for horizontal margins, only for deep margins
Differentiation	Poorly differentiated	Poorly differentiated	Poorly differentiated	No comment
Lymphovascular invasion (LVI)	LVI + (*intermediate risk*)	LVI +	LVI +	No comment
Depth of invasion	Haggitt 4 or Kikuchi Sm3	No comment	Submucosal invasion > 1 mm	> T1 depth
Tumour budding	Tumour budding present (intermediate risk)	No comment	Tumour budding present	No comment

The Japanese guidelines suggest that MPs can be endoscopically treated for any T1 disease, as long as technically feasible. JSCCR guidelines suggest that positive horizontal margin can be surveilled regularly for evidence of recurrence

The management of a patient diagnosed with a malignant polyp is a dilemma for clinicians. Traditionally, it was felt that as malignant polyps were a cancer, all patients should undergo a segmental lymphovascular colorectal resection, to remove the involved segment of bowel and lymph node basin. However, following segmental resection, only a minority of patients are proved to have residual disease in the bowel wall or metastatic spread to lymph nodes. Furthermore, colorectal surgery is associated with potentially significant complications including anastomotic leak, hospital acquired infections, sexual dysfunction, long-term changes to bowel habits and even death [12, 13]. Therefore, when confronted with the diagnosis of a malignant polyp, clinicians and patients must balance the risks of surgery against the likelihood of residual disease or metastatic spread.

### Aim

Whilst guidelines have been developed to suggest when clinicians should consider colorectal resection, this study sought to evaluate which patient pathological factors may influence the management strategy. The aim of this review was to evaluate the literature for the pathological and patient factors that predicted the management strategy for patients with malignant polyps—either colorectal resection or polypectomy with surveillance.

## Methods

This systematic review followed the methods and guidance of the Preferred Reporting Items for Systematic Reviews and Meta-Analyses (PRISMA) [14] and Cochrane Prognosis Methods Group [15] guidelines. This systematic review protocol was published in the PROSPERO Register for Systematic Reviews (CRD42021246504) on 13 May 2021 [16].

### Study design and participants

The population of interest were adult patients with a macroscopically complete endoluminal resection of an adenoma that contained a focus of submucosally invasive adenocarcinoma. Endoluminal resection was either via colonoscopic/endoscopic resection or by trans-anal excision of the polyp. Only studies which investigated patients with solitary, sporadic malignant polyps were included.

Exclusion criteria were patients with familial/inherited polyposis syndromes, inflammatory bowel disease, non-adenocarcinoma malignant polyps, polyps with intramucosal carcinoma (T0/carcinoma in situ), T2 or higher tumour stage, multiple malignant polyps or synchronous colorectal malignancy, previous history of colorectal cancer or post neoadjuvant therapy. Additionally, patients who had a malignant polyp that was not amenable to endoluminal resection for diagnosis were considered to have early invasive colorectal cancer and were also excluded. The landmark paper by Haggitt et al. in 1985 was one of the first to clarify the risk of lymph node metastases for malignant polyps [5] and effectively altered the management strategy of malignant polyps. Therefore, only papers published after 1985 were considered for this review.

### Systematic literature search

Following consultation with a professional university librarian, a search strategy was devised and performed on 14 May 2021. Web-based databases searched included PubMed, Embase, Scopus, Cochrane Database and Web of Science. The grey literature was not searched. Search terms included colonic neoplasms, rectal neoplasms, colorectal neoplasms, intestinal polyps, malignant, malignancy, malignancies and/or adenomas. Titles and abstracts of the literature search results were independently assessed by two authors (AZ and NL), and the relevant full texts were obtained. Full text article review was again completed independently by the same two authors. Any disagreements were resolved with discussion, and any disagreement was adjudicated by AR. Published abstracts alone were not included. The reference lists of the reviewed full text articles were appraised for further relevant studies. The risk of bias of the eligible studies was assessed with the QUality In Prognosis Studies (QUIPS) tool [17], using the phrase “management strategy” in place of “prognosis”, and “risk factor” replacing “prognostic factor” [17].

### Data extraction and outcomes

Recorded study information included study type, numbers of participants, participant selection method and the overall population size. The outcome recorded was the patient’s management strategy. All patient and pathological characteristics for each management strategy were recorded. Meta-analyses were performed using descriptive statistics from each study. Four studies performed statistical analysis using a multivariable model. Overall, the multivariable results appeared very similar to the univariate data; therefore, for consistency only the univariate data was included.

### Data synthesis

Random effects meta-analysis models were used to determine how management strategy was affected by the most commonly encountered variables namely: age, gender, polyp location, LVI, margin status, polyp morphology, tumour differentiation and depth of invasion as measured by Haggitt or Kikuchi levels [18]. The effect of categorical variables on management strategy was reported using odds ratios (OR) with 95% confidence intervals and 95% prediction intervals. The mean difference in age between management strategy groups was reported with a 95% confidence interval and a 95% prediction interval. For articles which reported no standard deviation [19, 20], estimation of standard deviation was calculated using the method described by Ma et al. [21]. For studies which did not publish a mean or standard deviation, the methods outlined by Wa et al. were applied to estimate a mean and standard deviation of age, assuming a normal distribution for age [22]. Heterogeneity was assessed using *I*^2^ and a *p* value. Analyses were performed using Stata v17.0 (STATA, College Station, TX, USA).

## Results

### Included and excluded studies

**Fig. 2:**
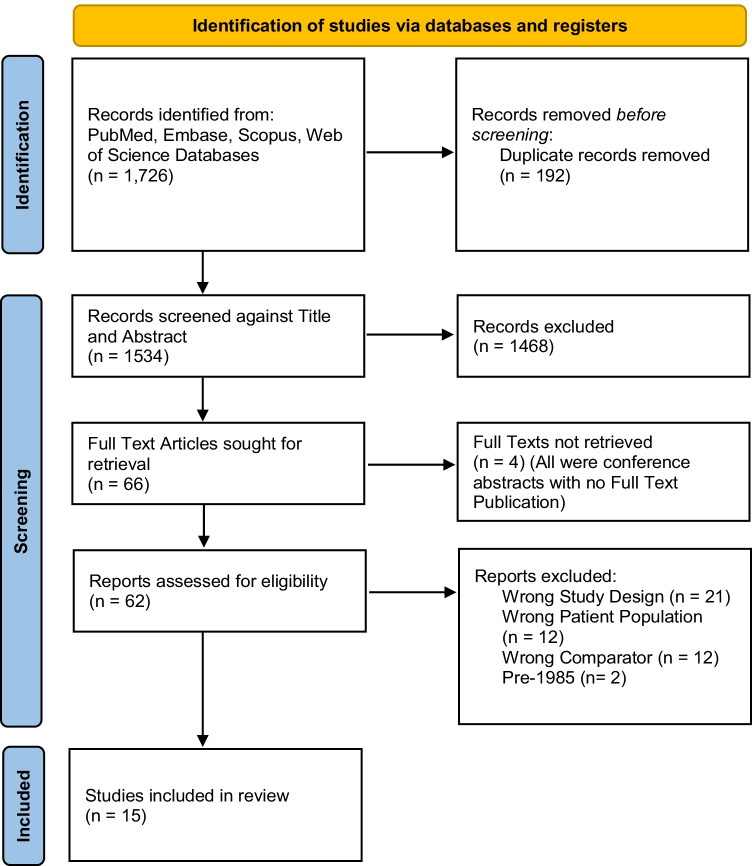
PRISMA diagram

Figure 2 is the PRISMA diagram summarising the outcome of the literature search and evaluation of studies. Initial literature search identified 1726 studies, 190 were duplicates and removed, a further 1467 were excluded after title and abstract review for not meeting the inclusion criteria of this meta-analysis, leaving 51 full texts that were reviewed. The majority of the 1467 papers excluded from full text review were papers which were investigating topics other than the management strategies of malignant polyps. Fifteen published studies comparing the differing characteristics of patients who were managed with either a polypectomy with surveillance or with surgical resection were identified [7, 19, 20, 23–34], and these studies are summarised in Table 2. Variables in each study were assessed and documented. The most commonly collected patient and polyp characteristics are documented in Table 3.

**Table 2:** Included studies

First author	Year published	Sample size	Study type	Patient selection
Brown et al. [34]	2016	239	Retrospective cohort study	All patients from private pathology database with a MP March 2007–September 2014 Total population size not applicable as from single pathology provider Moderate risk of bias for QUIPS Domain 1—given pathology only from private pathology database and may not represent general population
Cooper et al. [33]	2012	2077	Retrospective cohort study	All patients ≥ 66 years old with a MP diagnosed between 1992 and 2005. Using the Surveillance Epidemiology and End Results Medicare (SEER) Database Total population size not documented, only covers patients registered with Medicare—representing around 93% of the patients over 64 years old—and only in geographic areas part of the SEER programme Colonic only polyps included Moderate risk of bias for QUIPS Domain 1—given only colonic polyps included
Cunningham et al. [19]	1994	36	Not well described—likely case series	Unclear from methods. Methods state patients were identified retrospectively but do not give any further details on MP identification High risk of bias for QUIPS Domain 1—no description of participant selection
Fasoli et al. [32]	2015	306 (72 proceeded directly to surgery—suggesting these were not true MPs)	Retrospective cohort study	All MP detected in a colorectal cancer screening programme from April 2008 to April 2013 in 5 North-Eastern centres in Italy Total population size not documented
Fischer et al. [31]	2017	363	Retrospective cohort study	All MP from 5 out of 6 district health boards in New Zealand between 1999 and 2013 Total population size 2.25 million
Gill et al. [30]	2012	386	Retrospective cohort study	All MP from April 2006 to July 2010 from the NORthern Colorectal Cancer Audit Group (NORCCAG) database Total population size 3.1 million—all persons within the north of England
Gonçalves et al. [29]	2013	40	Retrospective cohort study	All MP from January 2007-November 2012 by a single department in a single hospital (Hospital Braga) Total population size N/A
Levic et al. [27]	2015	50	Retrospective cohort study	All MP from January 2003 to January 2008 from a single centre No documentation of total population covered
Levic et al. [28]	2019	692	Retrospective cohort study	All MP from the Danish Colorectal Cancer Group (DCCG) database, national pathology data bank and the Danish Patient registry Covering over 99% of Danish population, representing over 5.5 million people [36]
Netzer et al. [26]	1997	37	Retrospective cohort study	All MP from a single institution in St Gallen, Switzerland from 1986 to 1995 Hospital covered total population of 500,000
Senore et al. [25]	2018	392	Retrospective cohort study	All patients with a T1 colorectal cancer completely removed via endoscopy—essentially a MP. From 7 hospitals in North-Western Italy No documentation of total population covered
Sharma et al. [20]	2020	173	Retrospective cohort study	All patients with a MP from a single regional cancer network in UK from April 2012 to April 2015 Total population covered 1.5 million
Wasif et al. [7]	2011	19743	Retrospective cohort study	All MP from 1988 to 2003 identified in the SEER database. At the time this represented 26% of the US population Moderate risk of bias for QUIPS Domain 1—given colonic polyps, excluded rectal polyps
Whitlow et al. [24]	1997	59	Retrospective cohort study	All MP from 1972 to 1990 in a single institution (Oschner Clinic) with at least 6 months of follow up
Wu et al. [23]	2015	16	Retrospective case series	Case series of 16 patients with a sessile MP from a single endoscopist from 1997 to 2010 Moderate risk of bias for QUIPS Domain 1—may not be reflective of general population

**Table 3:** Most commonly collected variables

Variable	Number of studies reporting variable	Total number of patients reported
**Patient factors**		
Age (continuous variable)	8 (53%)	21827
Age (categorical variable)	3 (20%)	3320
Gender	9 (60%)	23763
Ethnicity	3 (20%)	21993
American Society of Anaesthesiology score	3 (20%)	994
Comorbidity scoring	4 (27%) NB: different scoring systems employed by different studies	924
**Polyp factors**		
Polyp location	11 (73%)	23977
Polyp morphology/type	10 (67%)	2050
Lymphovascular invasion	9 (60%)	913
Invasive cancer differentiation	8 (53%)	2224
Polyp size	7 (47%)	1291
Piecemeal resection	5 (33%)	1059
Haggitt/Kikuchi level	5 (33%)	538
Tumour budding	4 (27%)	353
Precursor polyp type	4 (27%)	20144
Mucinous differentiation	2 (13%)	931

### Risk of bias assessment

Whilst the QUIPS risk of bias tool is not a perfect fit for this series of meta-analyses, each domain from this tool was considered. For domain 1 (study participation), four studies were assessed as having a moderate risk of bias; a description of patient selection techniques is given in Table 2 [7, 23, 33, 34]. One study was rated at a high risk of bias as there was no description of patient selection techniques [19]. Domain 2 (study attrition), 3 (same measurement of predictors of management for the management strategies) and 4 (management measurement) were of no concern to the studies included in this meta-analysis. The effect of confounding (domain 5) was able to be partially assessed in the four studies which reported a multivariable model [7, 25, 30, 33]. In domain 6 (statistical analysis), five studies did not present descriptive statistics [19, 20, 23, 24, 26]; however, data from these studies were still included in the meta-analyses. Additionally, one other study did not fully report results for three pathological factors; these results are unlikely to affect the result of the meta-analyses (see below) [30].

### Meta-analysis results

#### Patient demographics

**Fig. 3:**
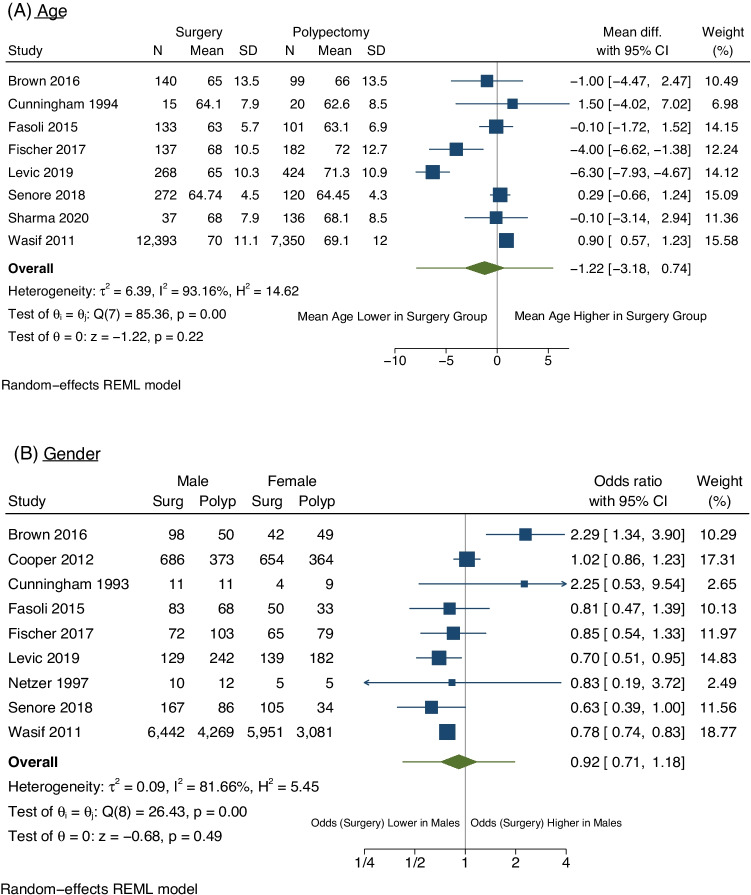
Meta analysis of patient factors that were investigated to predict management plan. The overall result with 95% confidence interval is shown by the green diamond, with the extending lines representing the 95% prediction interval. NB: Gill et al. [30], Levic et al. [28] and Cooper et al. [33] had data comparing age in a categorical format. Gill et al. [30] demonstrated odds of surgery in those < 70 was 2.18 times (95%CI: 1.22–3.87) that of those > 70 years old. Levic et al. [28] demonstrated with chi-squared statistic that management differed between those < 70 and > 70 (*p* < 0.001). Cooper et al. [33] demonstrated with chi-squared statistic that management strategy was significantly different amongst 5 different age groups (*p* < 0.001)

Four studies (21061 patients) included data on patient mean age and standard deviation (SD)/standard error (SEM) [7, 25, 28, 32]. The authors of the remaining four studies which reported age data were all contacted to obtain further information regarding the mean age and SD data. This information was received for one additional study [31]. Three studies had their mean and/or SD/SEM estimated [19, 20, 34]. Overall, eight studies (21827 patients) were included [7, 19, 20, 25, 28, 31, 32, 34]. The mean difference in age between the surgical management group and the polypectomy alone group was 1.22 years, which was not significant (95% CI − 0.72–3.42) (Fig. 3A). Nine studies (23763 patients) included patient gender [6, 8, 9, 19, 24, 32, 35–37]. The overall OR was 0.92 favouring the odds of surgical resection in males being lower than females; however, this was not significant (OR = 0.92, 95% CI 0.71–1.18) (Fig. 3B). Both age and gender analyses showed significant heterogeneity between studies, with *I*^2^ > 80% and *p* < 0.001 for heterogeneity in both analyses.

### Polyp characteristics

**Fig. 4:**
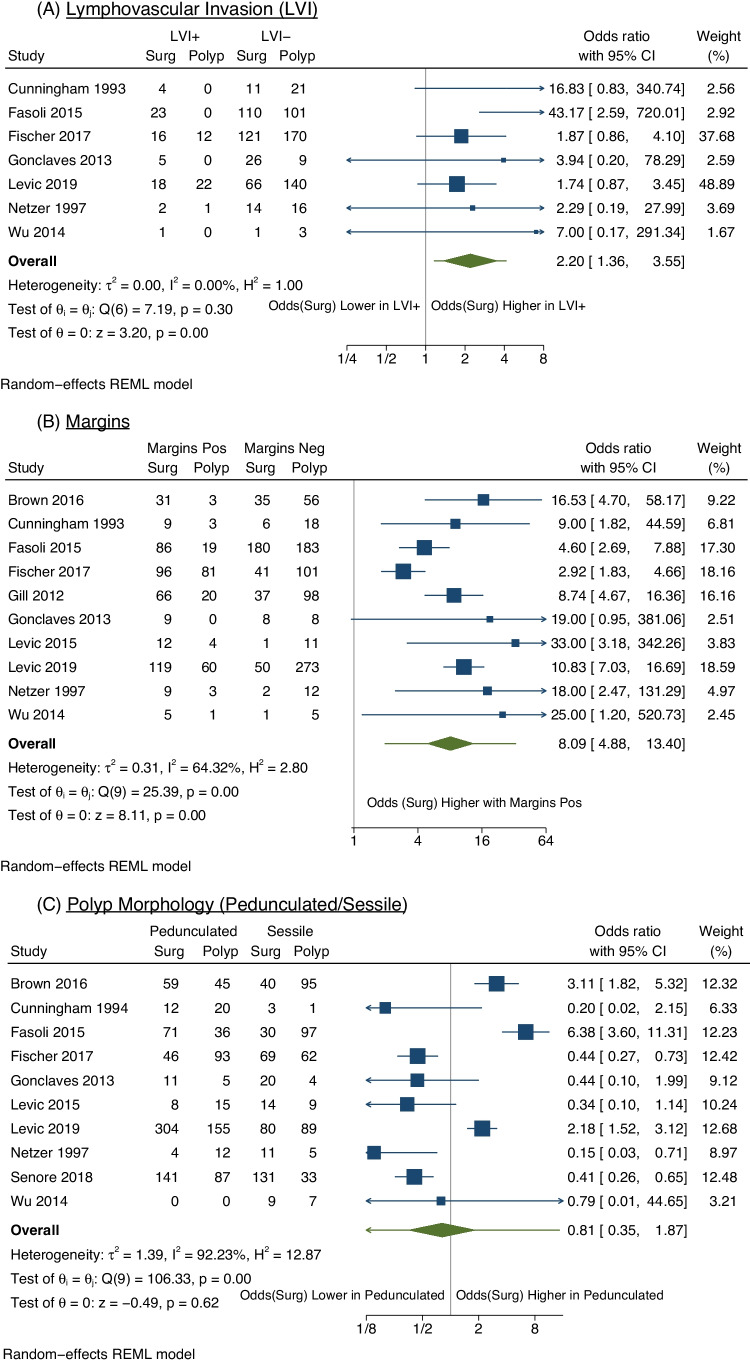

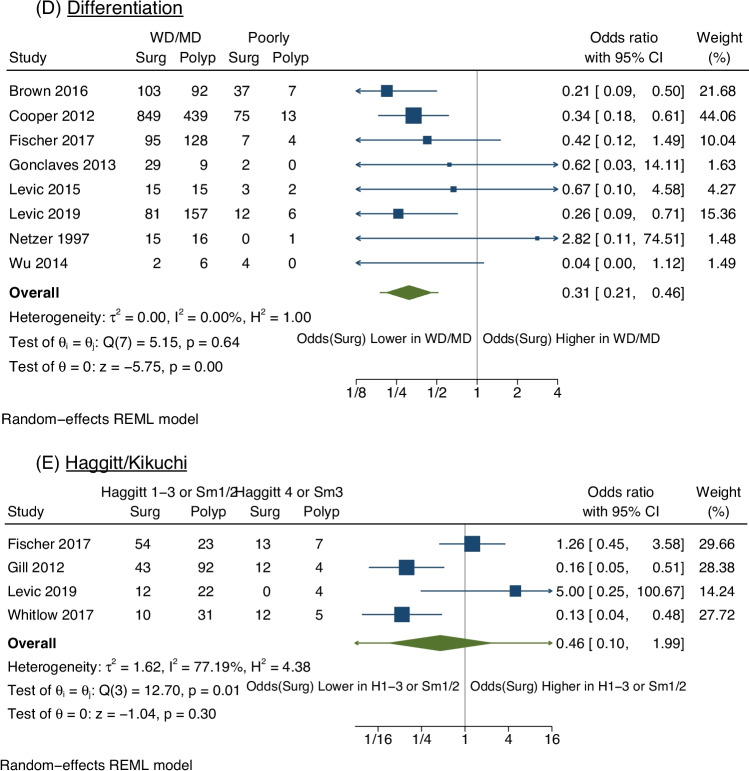
Meta analysis of polyp pathological factors that were investigated to predict management plan. Pathological factors that were all investigated by odds ratios with 95% confidence intervals were LVI (**A**), Margins (**B**), Polyp Morphology (**C**), Tumour Differentiation (**D**) and Haggitt/Kikuchi Levels (**E**). Margins Pos indicates involved or margins within 1 mm. WD/MD indicates cancers that were well or moderately differentiated; poorly indicates cancers that were poorly differentiated. H1–3 indicates patients with Haggitt levels 1–3. Surg/Surgery indicates patients who were managed with a colorectal resection, whilst Polyp/Polypectomy indicates patients who were managed with polypectomy with surveillance. NB: Gill et al. [30] reported that LVI, differentiation and polyp morphology were not found to influence management strategy significantly. However, Gill et al. did not present their summary data, and so the study was not included these meta-analyses. Estimating the raw data, with an odds ratio of 1, did not significantly influence the outcomes of these meta-analyses

Seven studies (913 patients) included the presence of tumour LVI when reporting the management plan [19]. There were significantly higher rates of surgery for patients with polyps with LVI (OR 2.20, 95% CI 1.36–3.55) (Fig. 4A). There was negligible heterogeneity (*I*^2^ = 0%, *p* = 0.3).

Ten studies (1762 patients) evaluated positive or close margins [19, 23, 26–32, 34]. There were differing definitions of positive margin status; therefore, to maximise the numbers of patients included in the statistical analysis, positive margins were considered together with close margins, meaning the presence of tumour cells within 1 mm of the polypectomy margin was included together. This definition is consistent with management guidelines [6]. The rate of surgery was significantly higher for those with positive or close margins (OR 8.09, 95% CI 4.88–13.40) (Fig. 4B). There was a moderate degree of heterogeneity (*I*^2^ = 64%, *p* = 0.003).

Ten studies (1933 patients) evaluated the type of polyp (pedunculated vs sessile) [19, 23, 25–29, 31, 32, 34]. The pooled odds ratio was not significant when comparing patient management based upon whether the polyp was pedunculated or sessile (OR 0.81, 95% CI 0.35–1.87) (Fig. 4C). There was a high degree of heterogeneity amongst studies (*I*^2^ = 92%, *p* < 0.001).

Eight studies (2224 patients) included data on tumour differentiation [23, 26–29, 31, 33, 34]. Well/moderately differentiated tumours were compared to poorly differentiated tumours. The overall odds of surgery were significantly lower in those with well differentiated or moderately differentiated tumours compared to poorly differentiated tumours (OR 0.31, 95% CI 0.21–0.46) (Fig. 4D). There was negligible heterogeneity (*I*^2^ = 0%, *p* = 0.64).

Whilst five studies assessed Haggitt or Kikuchi levels, one study grouped Haggitt/Kikuchi levels differently [34], and inclusion in the meta-analysis was not possible for this study. Across the remaining four studies (344 patients) [24, 28, 30, 31], a trend towards polypectomy and surveillance for patients with Haggitt level 1–3 or Kikuchi Sm1-2 level invasion was observed but was not statistically significant (OR 0.46, 95% CI 0.10–1.99) (Fig. 4E). There was a high degree of heterogeneity (*I*^2^ = 77%, *p* = 0.005).

Mucinous differentiation was only reported in 2 studies, precluding its assessment in a meta-analysis [28, 34]. Likewise, tumour budding was only reported in 4 studies [27, 28, 32, 34], with one study only reporting budding status for a single patient [27]; thus, tumour budding was not assessed in a meta-analysis.

## Discussion

These meta-analyses assessed patient and pathological factors which predicted patient management. This is the first series of meta-analyses directly assessing the factors which predict the management strategy for malignant colorectal polyps. The key findings were that LVI, close or positive margins and poorly differentiated tumours all had a statistically significant higher odds of proceeding to surgery over polypectomy and surveillance. Conversely, age, gender, polyp type and a Haggitt/Kikuchi assessment of depth of invasion did not have statistically significantly different odds of proceeding to surgery post polypectomy.

This review investigated four of the six pathological factors, suggested by the ACPGBI guidelines, which may affect the management strategy. LVI, poor differentiation and close or involved tumour polypectomy margins were all significant predictors of resection. The presence of LVI in a polypectomy specimen has previously been reported to be associated with an increased risk of metastatic lymphatic disease [38]. Likewise, poor differentiation has been associated with an increased risk of residual disease and lymphatic spread [4]. Finally, close or positive margins present a logical risk of residual disease [6]. Thus, LVI, poor differentiation and close or involved polypectomy margins all increase the risk of residual or metastatic disease, and ACPGBI guidelines suggest colorectal resection as the appropriate management strategy. These meta-analyses suggest that the ACPGBI recommendations are being followed in practice [6].

Whilst clinical guidelines suggest patients with a Haggitt level 4 or Kikuchi level Sm3 malignant polyps should be considered for resection, this was not statistically reflected in this meta-analysis. However, it must be noted that this pathology detail was not commonly recorded and represented only a minority of the total patient cohort across all studies. Furthermore, even in studies which contained data on Haggitt/Kikuchi levels, there were large numbers of study patients without a Haggitt or Kikuchi level recorded [28]. There may be pathological reasons that Haggitt or Kikuchi levels were unable to be included in histopathological reports. These may include incomplete polypectomy, fragmented polypectomy specimens and difficulty determining polyp morphology when ex situ. It is noted that pathological reporting is increasingly moving towards quantitative reporting of depth of invasion in millimetres, either in addition to or instead of a Haggitt or Kikuchi level [10]. Future studies investigating management decisions for malignant polyps should consider collecting this direct measure of depth of invasion. Of the fifteen studies in this meta-analysis, only one contained this measurement [34]. Depth of invasion, whether measured as a Haggitt/Kikuchi level, or as a direct measure of invasion below the muscularis mucosae has been significantly associated with lymphatic and residual disease [5, 8, 38, 39]. However, depth of invasion may not always be reported in an interpretable format in pathological reporting, and this was highlighted in this meta-analysis. It is likely that the lack of this information in the published literature, reflects current clinical practice and histopathological reports. As depth of invasion plays an important role in estimation of the risk of residual or lymphatic disease, clinicians may be warranted in insisting on an estimate of depth of invasion, to provide patients with the best estimate of risk of residual or lymphatic disease.

The ACPGBI scoring system for determining a recommended management strategy for malignant polyps also considers the pathological factors of the presence of mucinous differentiation and tumour budding [6]. Both mucinous differentiation and tumour budding were unable to be assessed in this meta-analyses due to the small number of studies including these pathological parameters.

When comparing demographic characteristics, there were no significant differences in the age or gender of patients with malignant polyps when comparing polypectomy alone with surgical management. It would be anticipated that older patients would be at higher risk of surgical morbidity and mortality, and might be less likely to proceed to surgery. Despite the trend towards a younger age for patients undergoing surgery, this was not statistically significant.

There existed significant heterogeneity within some of the meta-analyses that were performed in this review (Figs. 3 and 4). Since 1985 there have been significant changes in patient expectations, operative techniques and anaesthetic practice. The variable adoption over time of these practices across the different surgical departments have likely contributed significantly to the heterogeneity seen in these meta-analyses (Table 2).

One limitation in this series of meta-analyses was the inability to compare an overall risk of individual malignant polyps between the polypectomy and surgical resection groups. There is the potential that some studies could have featured a high number of patients with a single high-risk pathological factor which encouraged clinicians to consider colorectal surgical resection. As such other high-risk pathological factors may not have been present, yet the patient still proceeded to surgery, potentially introducing a source of bias in these results. However, it is noted that often high-risk features for residual or metastatic disease appear concurrently, such as greater depth of invasion and LVI [40] and so reducing this potential of bias. The ACPGBI guidelines combine all risk factors into a summary score of risk. No study identified in this review directly compared the ACPGBI risk categories between management strategies. Future research should consider using a known published risk categorisation tool, such as the one published by the ACPGBI to reduce this risk of bias. Another limitation of this review was the diverse patient and polyp factors collected or reported. Guidelines clearly document indications for clinicians to consider surgical resection; however, this study found that few patient characteristics were reported to understand why patients underwent surveillance, when guidelines would recommend surgical management. It would have been of interest to evaluate patient comorbidities that may influence patient selection for surgical resection. A surrogate of patient comorbidities is the American Society of Anaesthesiologists (ASA) Score, which grades patients based upon their pre-operative comorbidities [41]. Thus, it was considered whether a patient ASA could be assessed in a meta-analysis. However, the ASA was only reported in three studies, thus limiting the ability to analyse this important determinant.

Further research should focus on understanding the decision-making process to arrive at polypectomy and surveillance versus surgery. Whilst LVI, margin status and poor differentiation were shown to be a predictor of surgery, there were still a number of patients with these pathological features, who did not undergo surgical resection. Patient choice, comorbidities or clinician lack of familiarity with malignant polyp guidelines may explain why these patients did not undergo resection. Pathological factors associated with increased risk of lymphatic spread, such as Haggitt or Kikuchi levels, were commonly not reported. The reasons for this under-reporting would be of interest to further research and quality improvement activities. Further strategies to educate clinicians and patients on their risk of residual or metastatic disease, in the setting of a malignant polyp, may be helpful. Lastly, comparison of overall malignant polyp risk, in the comparison of ACPGBI risk categories, between management strategies would reduce the risk of bias in future studies.

## Conclusion

The management of malignant polyps remains a treatment dilemma. This is the first known meta-analysis evaluating the factors guiding the management strategy for patients with malignant polyps. From the fifteen studies evaluated, the presence of LVI, positive or close margins and poorly differentiated tumours were all significantly associated with patients undergoing a colorectal segmental resection. Whilst Haggitt/Kikuchi level was not statistically significantly associated with patients undergoing a colorectal resection, this assessment was limited by high heterogeneity and limited numbers in the studies assessed. Further population-based analysis would assist in understanding the actual management preferences and adherence to guidelines for malignant polyps in the wider community.
